# A bibliometric analysis of exosomes in aging from 2007 to 2023

**DOI:** 10.3389/fmed.2024.1488536

**Published:** 2025-01-22

**Authors:** Zenghui Niu, Meiyu Cui, Yingkun Fu, Lingfeng Zhou, Jiali Wang, Yan Lei, Xinrong Fan, Qiang Wang, Jing Yang

**Affiliations:** ^1^Beijing Key Laboratory of Traditional Chinese Medicine Basic Research on Prevention and Treatment for Major Diseases, Experimental Research Center, China Academy of Chinese Medical Sciences, Beijing, China; ^2^Guanganmen Hospital, China Academy of Chinese Medical Sciences, Beijing, China; ^3^Tianjin Academy of Traditional Chinese Medicine Affiliated Hospital, Tianjin, China; ^4^Wangjing Hospital, China Academy of Chinese Medical Sciences, Beijing, China

**Keywords:** bibliometric, exosomes, aging, senescence, aging-related diseases

## Abstract

**Background:**

Aging is the primary factor contributing to the development of aging-related diseases. As research on exosomes continues to advance, its relationship with aging and aging-related diseases has become a hot topic This article analyzes the research hotspots of exosomes in aging and aging-related diseases, aiming to fill the gap in bibliometric research in this field and help researchers better understand the current status and future trends of both fundamental and clinical research in this field.

**Methods:**

The articles were retrieved and exported from WoSCC on December 18, 2023. The visual analysis of countries and regions, institutions, authors, references, and keywords in exosomes of aging was conducted using VOSviewer 1.6.18, CiteSpace 6.2.R7, and Bibliometrix.

**Results:**

The bibliometric analysis included 1628 articles. China and the United States emerged as the top two leading countries in this field. A total of 2,321 research institutions from 78 countries and regions were primarily led by China and the United States. Both Kapogiannis D and Goetzl E were active authors in this field. Thery C, Valadi H, and Raposo G were the important promoters in this field. Thery C proposed the method of differential centrifugation and density gradient centrifugation to extract exosomes. Valadi H discovered cells could send RNA-messages to each other by loading them into exosome-vesicles. The journal with the highest number of articles was *International Journal of Molecular Sciences*, while *PLoS One* was the most frequently cited journal. The keyword analysis revealed that future research on exosomes in aging will possibly focus on “inflammation, cellular senescence, angiogenesis, insulin resistance, and Alzheimer's disease.”

**Conclusion:**

We identified the research trends of exosomes in the field of aging through this bibliometric analysis. The present study provides valuable new perspectives on the history and current status of exosomes in the field of aging and aging-related diseases, and also offering guidance for future research directions.

## 1 Introduction

Aging is an inevitable process that organisms spontaneously undergo over time, manifesting structural and functional degeneration, loss of adaptability and resistance. Concurrently, aging is an inevitable and complex phenomenon characterized by a variety of factors, including genomic instability, telomere shortening, epigenetic modifications, mitochondrial dysfunction, cellular senescence, alterations in intercellular communication, and inflammatory responses (1). Despite outstanding progress in the medical research of aging risk factors, aging-related diseases remain the leading cause affecting health issues in humans.

Exosomes are cell-secreted vesicles of a lipid bilayer membrane with a diameter about 30~150 nm (the average diameter is about 100 nm), and they are one of the important carriers for regulating intercellular communication. Furthermore, exosomes influence the senescence-associated secretory phenotype (SASP) in aging and aging-related diseases. Juan Antonio Fafian-Labora demonstrated that exosomes isolated from young fibroblasts reduce tissue damage by decreasing oxidative stress and lipid peroxidation (2). Another study showed that exosomes derived from bone marrow mesenchymal stem cells (MSCs) play a role in regulating nucleus pulposus cell senescence (3) and aging-related insulin resistance (IR) (4). Besides that, exosomes can also regulate the microenvironment and induce cellular senescence through autocrine or paracrine pathways. Hadi Valadi discovered that exosomes deliver mRNAs and miRNAs to new cells and play a functional role within these cells (5). Another study showed that exosomes spread to the surrounding environment and induce aging in young cells (6). What's more, miRNAs have been recognized as the biomarkers of certain diseases and pathological states, such as exosomal miR-24-3p in the saliva of elderly individuals (7). Therefore, it is necessary to explore the mechanisms of aging and prevention and treatment of diseases to analyze the relationship between exosomes and aging.

**Figure 1:**
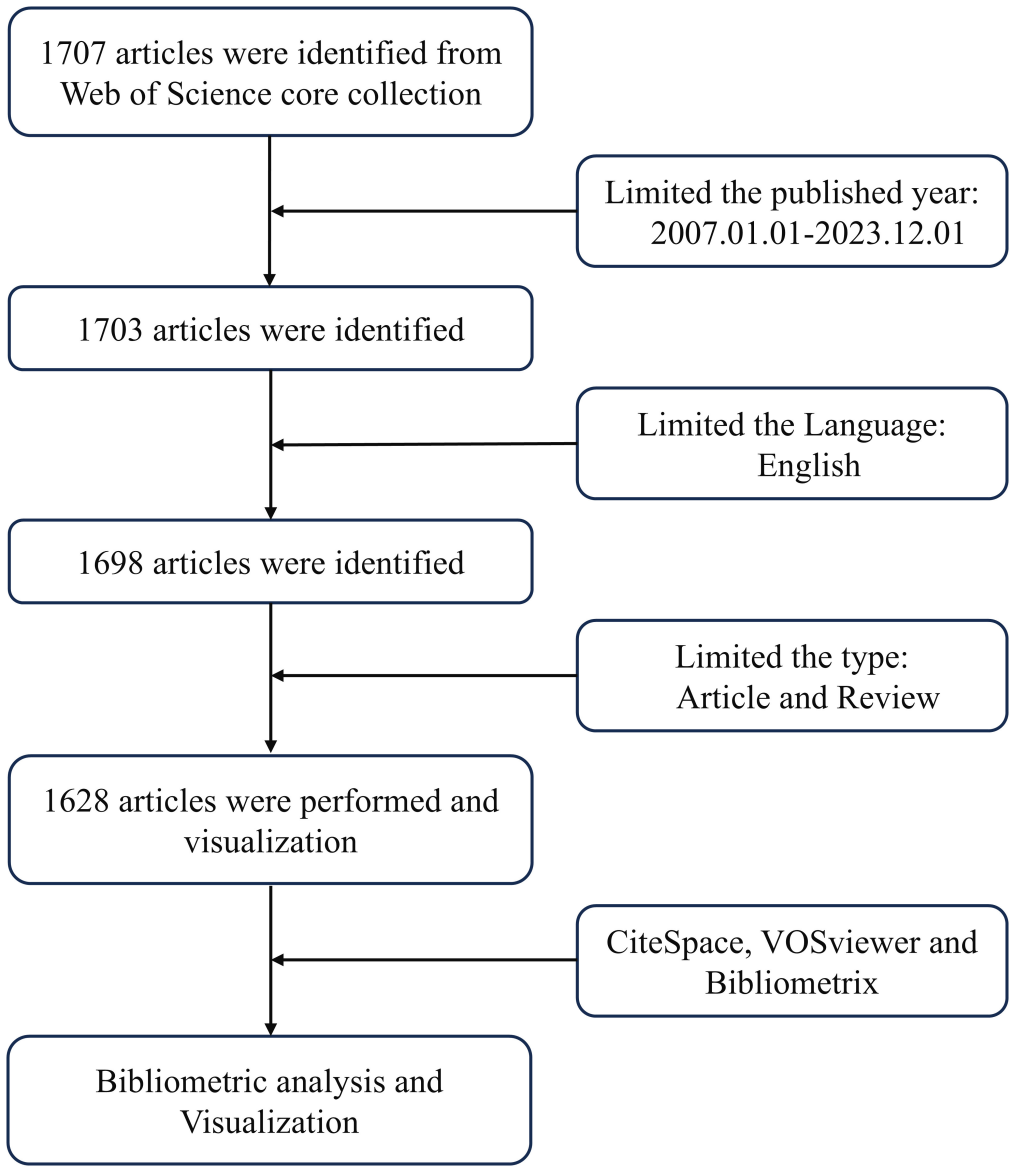
Search strategy schematic.

The bibliometrics emerged as an independent discipline in 1969 (8), which is a quantitative approach to the existing studies in a particular field and time period (9). CiteSpace describes the evolution of research fields and the historical development of clustering from a temporal perspective (10). VOSviewer provides network, overlay, and density, visualizations for keywords, and other parameters to build visual network maps (11–14). Bibliometrix allows for rapid integration and upgrading with other statistical and graphical tools in bibliometric analysis (15). Through bibliometric analysis, we can not only deeply explore the relationship between a thesis and its information, but also predict the development of a field. However, bibliometrics has not been used to investigate exosomes in aging. Therefore, in this study, we used bibliometric methods, searched the Web of Science Core Collection (WoSCC), and explored the hotspots and development trends of exosomes in the field of aging and drew a map of scientific knowledge in order to support intercellular communication-based aging research (Figure 1).

## 2 Materials and methods

### 2.1 Data collection

The literature was retrieved from WoSCC, which is regarded as the most appropriate database for high-quality digital bibliometric resources (16). All literature was downloaded in plain text format within 1 day, on December 18, 2023. The search themes were as follows: “TS = (aging OR senescence),” AND “TS = (exosomes),” AND “Reference Type: Article AND Review, AND Language: English,” and the retrieval date range was from January 1, 2007, to December 1, 2023. A bibliometric analysis was conducted on a total of 1,628 selected works of literature.

### 2.2 Data analysis

All WoSCC standards-compliant data was imported into VOSviewer 1.6.18, CiteSpace 6.2.R7 and Bibliometrix for literature visualization analysis. Bibliometrix shows the evaluation results for metadate in our data (Supplementary Figure 1). CiteSpace, VOSviewer and Bibliometrix were used to analyze the visual distribution of countries and regions, authors and co-cited authors, journals and co-cited journals, co-cited references, keywords cluster analysis, and timelines.

## 3 Results

### 3.1 Keywords and timezone map

**Figure 2:**
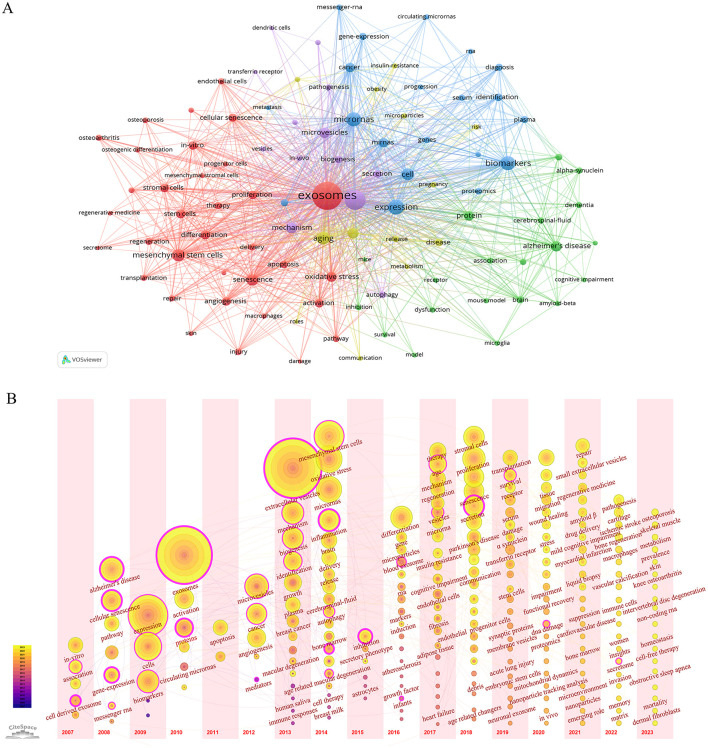
**(A)** VOSviewer visualization map of keywords clustering analysis on exosomes in aging. **(B)** CiteSpace visualization map of timezone viewer related to exosomes in aging.

This research involved a total of 6698 keywords. In Table 1, miRNAs were the most frequently used keyword in exosome-related aging research, followed by biomarkers MSCs, mechanism, and microvesicles. MiRNAs and biomarkers had appeared more than 250 times among these keywords, indicating that these research fields were hotspots and might have substantial research potential. In Supplementary Figure 2, extracellular vesicles and miRNAs were closer to red, representing their higher density and indicating that the keyword was a research focus and hotspot in the field, while the other parts closer to blue indicated that the field was not currently receiving extensive attention. Figure 2A and Supplementary Table 1 depicted five different clusters representing five different directions. Figure 2B presented a timezone map categorizing keywords. The first stage was from 2007 to 2012, the primary keywords were cellular senescence, expression, apoptosis, biomarkers, and so on. The field of aging was beginning to look at exosome-related research, focusing on apoptosis, gene expression, RNA, and related diseases such as Alzheimer's disease (AD) and cancer. Most of the research themes generated during this period continue to be researched and advanced to this day. From 2013 to 2017, the second phase focused on MSCs, oxidative stress, inflammation, therapy. The regenerative ability of MSCs had received widespread attention, and the research on the mechanism of exosomes interfering with aging and aging-related diseases was continuously deepened. The third stage was from 2018 to 2023, mainly focusing on stromal cells, proliferation, Parkinson's disease, communication, transplantation, regenerative medicine. This stage was characterized by the intensification of research fields and the expansion of research topics. In recent years, several prominent research areas have emerged, notably osteoporosis, skeletal muscle, knee osteoarthritis, obstructive sleep apnea, and cell-free therapy.

**Table 1:** Top 20 Keywords on “exosomes in aging.”

**No**.	**Keywords**	**Count**	**No**.	**Keywords**	**Count**
1	Exosomes	1,160	11	Microvesicles	141
2	Extracellular vesicles	541	12	Inflammation	135
3	Expression	296	13	Alzheimer's disease	134
4	MicroRNAs	258	14	Oxidative stress	124
5	Biomarkers	255	15	Protein	111
6	Aging	209	16	Cancer	107
7	Mesenchymal stem cells	190	17	Stem cells	101
8	Cell	170	18	Differentiation	98
9	Senescence	159	19	Proliferation	89
10	Mechanism	147	20	Stromal cells	89

### 3.2 The trend of publication outputs

**Figure 3:**
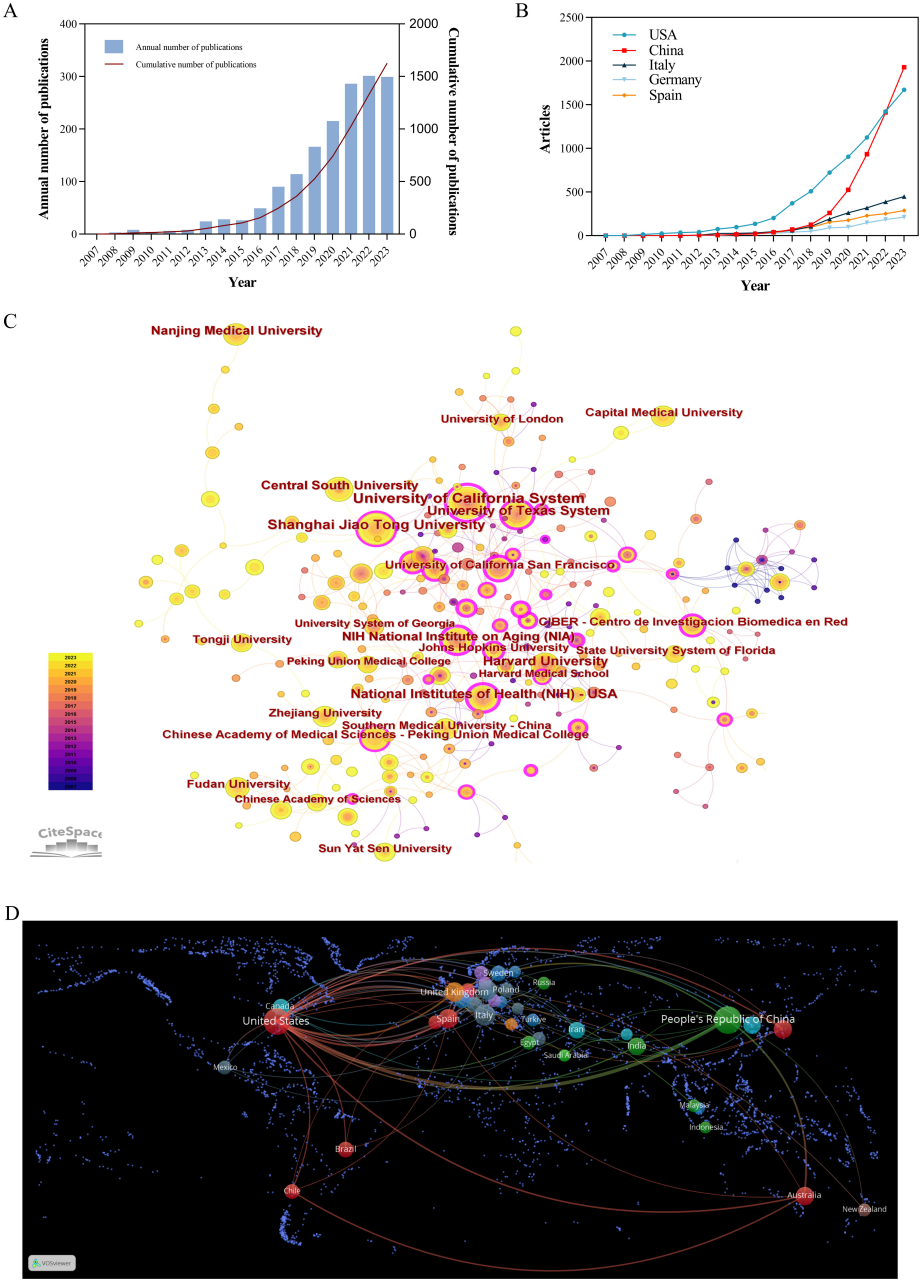
**(A)** Time trend of the publications on exosomes in aging from 2007 to 2023. **(B)** Distribution of publications from different countries. **(C)** CiteSpace visualization map of institutions involved in exosomes in aging. **(D)** Map of cooperation between countries.

A total of 1,628 publications were obtained from the WoSCC, comprising 1,222 articles and 406 reviews. As depicted in Figure 3A, the fewest published articles were recorded in 2007, with only 1 article, whereas the highest count was 301 articles in 2022. From 2007 to 2012, research on exosomes in aging was in its early stages, with 30 articles. The second phase spanned from 2013 to 2017, during which 217 articles were published. The final stage spanned from 2018 to 2023, during which the annual number of articles consistently exceeded 100. From 2020 to 2023, the annual number of publications exceeded 200, indicating a substantial increase compared to that in 2019. In the past 6 years, 1,381 articles were published, accounting for 84.83% of the overall publications. This trend underscores a growing momentum in research activities related to the correlation between exosomes and the aging process.

### 3.3 Distribution of countries/regions and institutions

This research encompassed a total of 2,321 institutions across 78 countries and regions. As shown in Figure 3B and Table 2 most articles came from China (524, 32.19%), and the US (490, 30.1%). The University of California System from the United States had emerged as the leading research institution with the highest number of published articles (54 articles, 3.32%), closely followed by Shanghai Jiao Tong University from China (49 articles, 3.01%). Among the top ten institutions based on the quantity of published articles, there are five institutions in the United States and China.

**Table 2:** Publications of the top ten countries and institutions.

**NO**	**Country**	**Centrality**	**Count (%)**	**Institution**	**Centrality**	**Count (%)**
1	Peoples R China	0.12	524 (32.19%)	University of California System (US)	0.18	54 (3.32%)
2	US	0.58	490 (30.10%)	Shanghai Jiao Tong University (China)	0.15	49 (3.01%)
3	Italy	0.18	127 (7.80%)	NIH (US)	0.10	39 (2.40%)
4	Spain	0.08	78 (4.79%)	University of Texas System (US)	0.13	35 (2.15%)
5	Germany	0.17	73 (4.48%)	Harvard University (US)	0.15	32 (1.97%)
6	Japan	0.06	66 (4.05%)	NIA (US)	0.04	32 (1.97%)
7	Australia	0.04	63 (3.87%)	Central South University (China)	0.01	31 (1.90%)
8	England	0.13	62 (3.81%)	Nanjing Medical University (China)	0.01	29 (1.78%)
9	South Korea	0.01	51 (3.13%)	Chinese Academy of Medical Sciences - Peking Union Medical College (China)	0.04	27 (1.66%)
10	India	0.12	46 (2.83%)	Fudan University	0.01	26 (1.60%)

Betweenness centrality (BC) values above 0.1 indicate pivotal nodes. In Table 2, several countries exhibited a higher degree of centrality compared to others such as the United States (0.58), Italy (0.18), and France (0.18). The line between the circles indicated the cooperative relationship between countries and institutions. In Figures 3C, D, the United States had developed extensive cooperative relationship with the United Kingdom, Netherlands, Spain, Australia, Brazil, China, Japan, and Korea. Additionally, the University of California System maintained collaborative partnership with the US Department of Veterans Affairs, the University System of Georgia, the University of Miami, Veterans Health Administration (VHA), the University of Texas System.

**Figure 4:**
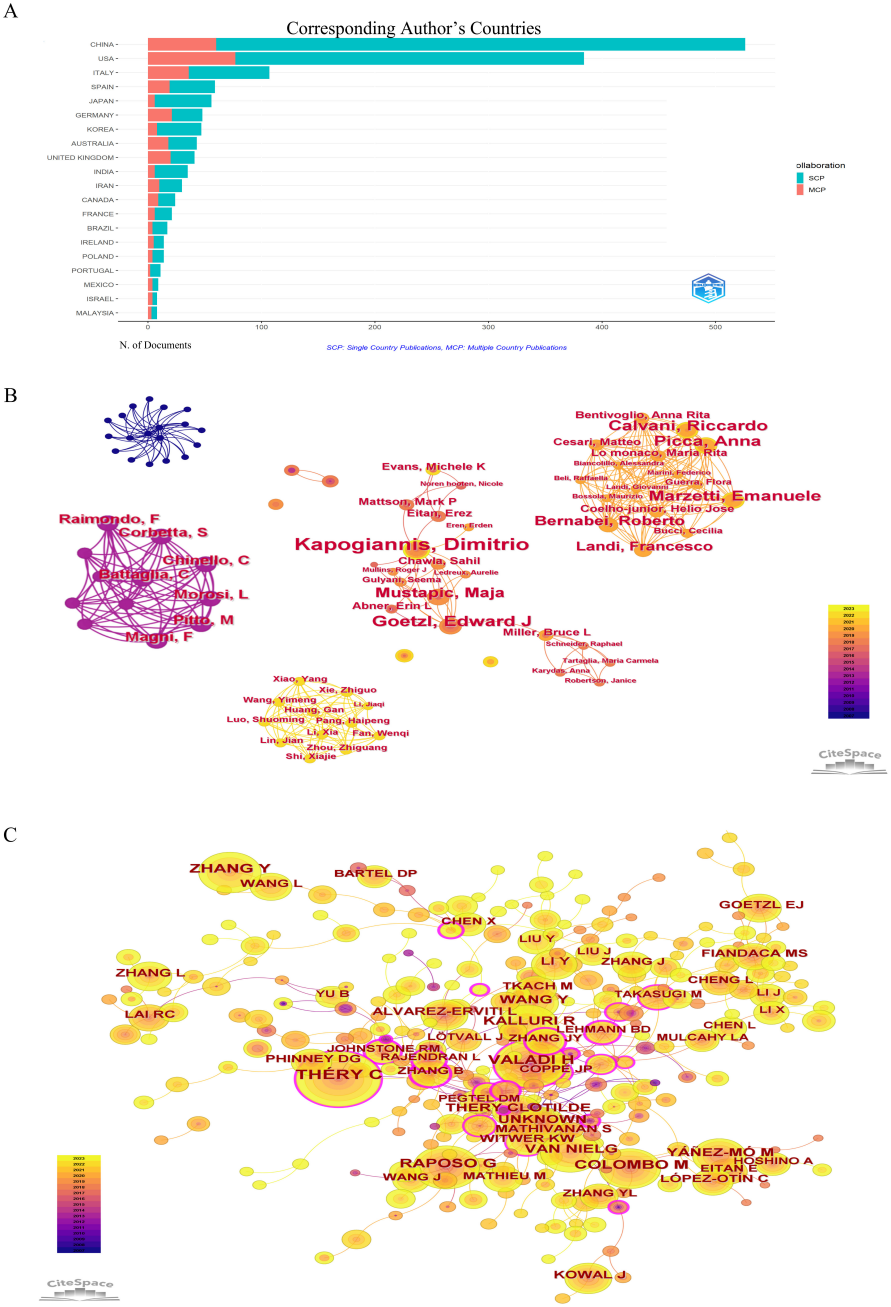
**(A)** Cooperation in the country where the corresponding author is located. **(B)** CiteSpace visualization map of authors involved in exosomes in aging. **(C)** Co-cited authors involved in exosomes in aging.

However, most countries/regions and research institutions were not sufficiently focused and lacked stable and in-depth communication and collaboration. For example, in Figure 3B and Table 2, China had published 524 articles on exosomes in aging, but its Centrality is only 0.12. The United States had the second highest number of publications (490, 30.1%), but it had a high centrality (0.58). As shown in Figure 3D, Supplementary Figures 3, 4, the number of international cooperation nodes in the United States was significantly higher than that of China. Although China had initially formed a national cooperation network, there was still a need to continue to expand the scope and depth of international cooperation. Meanwhile, in Figure 4A, the number of MCP publications in the countries of the corresponding authors in the United States was more than that in China. Therefore, it was also important to emphasize international cooperation and shared research results to promote scientific progress while we focused on research.

### 3.4 Authors/journals and co-authors/journals

A total of 10,659 authors had contributed to articles on exosomes in aging. As shown in Table 3, Kapogiannis D was the most published author (17 articles, 1.04%), the second position was occupied by Goetzl E (11 articles, 0.68%). Each node represented an author, and the larger the node, the greater the number of articles published by that author. Thicker lines represented greater collaboration between authors. Differently colored connection nodes represented cooperative clusters among different authors. According to Figure 4B, authors established a network of communication and collaboration. Co-cited authors were two or more authors who were cited together in the same paper or in multiple articles (Figure 4C). As shown in Supplementary Table 2, Thery C (339, 0.18) was the most cited author, followed by Valadi H (230, 0.06), Raposo G (200, 0.05), and Zhang Y (179, 0).

**Table 3:** TOP 10 authors related to exosomes in aging.

**NO**	**Authors**	**Count**	**H-index**	**Articles**
1	Kapogiannis, Dimitrios	17	52	Association of Extracellular Vesicle Biomarkers With Alzheimer Disease in the Baltimore Longitudinal Study of Aging
2	Goetzl, Edward J	11	106	High complement levels in astrocyte-derived exosomes of Alzheimer disease
3	Marzetti, Emanuele	10	74	Inter-Organelle Membrane Contact Sites and Mitochondrial Quality Control during Aging: A Geroscience View
4	Calvani, Riccardo	10	55	Mitochondrial dysfunction and aging: insights from the analysis of extracellular vesicles
5	Picca, Anna	10	43	A novel multi-marker discovery approach identifies new serum biomarkers for Parkinson's disease in older people: an Exosomes in Parkinson Disease (EXPAND) ancillary study
6	Liu, YutaoPicca, Anna	10	28	Emerging role of extracellular vesicles in musculoskeletal diseases
7	Mustapic, Maja	9	32	Plasma extracellular vesicles enriched for neuronal origin: a potential window into brain pathologic processes
8	Menon, Ramkumar	8	60	Amnion epithelial cell-derived exosomes induce inflammatory changes in uterine cell
9	Bernabei, Roberto	8	90	Adults with Physical Frailty and Sarcopenia Show Increased Levels of Circulating Small Extracellular Vesicles with a Specific Mitochondrial Signature
10	Landi, Francesco	7	100	Circulating amino acid signature in older people with Parkinson's disease: A metabolic complement to the EXosomes in PArkiNson Disease (EXPAND) study

*H-index is from WoSCC*.

A total of 697 academic journals had published articles on exosomes in aging. As shown in Table 4, *International Journal of Molecular Sciences* had published the highest number of articles (71 articles), followed by *Cells* (36 articles), *Scientific Reports* (31 articles), and *Stem Cell Research & Therapy* (29 articles). The journal with the highest impact factor among the top 10 was *Journal of Extracellular Vesicles* (IF: 15.5). In the co-cited journal of 6,738, 32 reference number more than 500 times, and 11 journals had been cited over 1,000 times. Table 4 revealed that the top 10 journals had been cited more than 1,000 times. *Natur*e had the highest impact factor among the top 10 literature, followed by *Cell* (IF: 45.5), *Nature Communications* (IF: 14.7), and *Journal of Extracellular Vesicles* (IF: 16).

**Table 4:** Top 10 journals and co-cited journals related to exosomes in aging.

**NO**	**Journal**	**Count**	**IF**	**Co-cited journal**	**Co-citations**	**IF**
1	International Journal of Molecular Sciences	71	4.9	PLoS One	2791	2.9
2	Cells	36	5.1	Proceedings of the National Academy of Sciences of the United States of America	1910	9.4
3	Scientific Reports	31	3.8	Scientific Reports	1901	3.8
4	Stem Cell Research & Therapy	29	7.1	International Journal of Molecular Sciences	1800	4.9
5	PLoS One	27	2.9	Journal of Biological Chemistry	1747	4
6	Frontiers in Cell and Developmental Biology	27	4.6	Journal of Extracellular Vesicles	1686	15.5
7	Frontiers in Immunology	24	5.7	Nature	1532	50.5
8	Journal of Extracellular Vesicles	17	15.5	Stem Cell Research & Therapy	1450	7.1
9	Aging-US	16	3.9	Cell	1414	45.5
10	Frontiers in Endocrinology	15	3.9	Nature Communications	1054	14.7

*Impact Factor (IF) comes from Journal Citation Reports of WoSCC*.

### 3.5 Co-cited references and references burst

**Figure 5:**
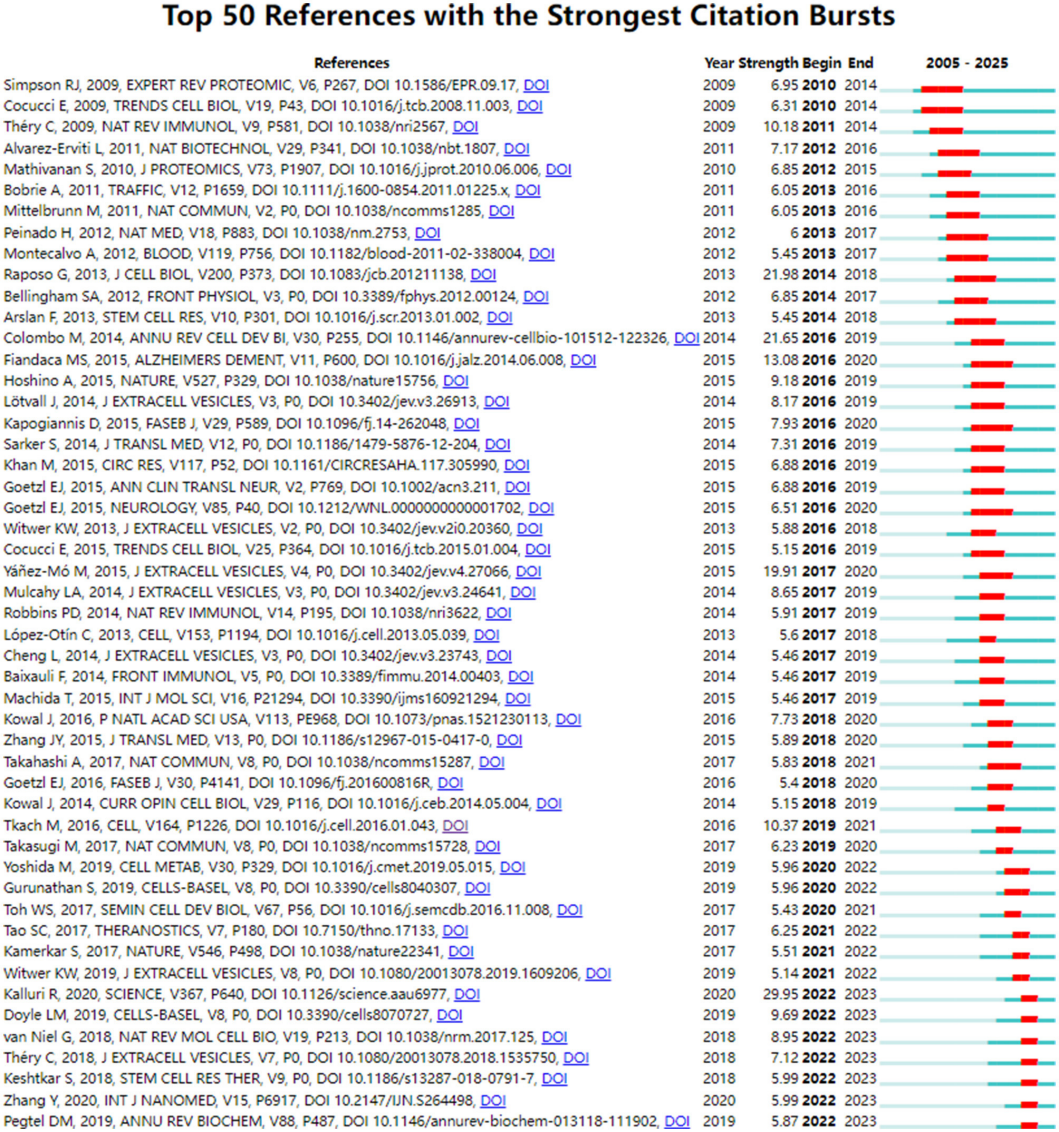
CiteSpace visualization map of top 50 references with the strongest citation bursts involved in exosomes in aging.

Through co-citation analysis, this study identified research hotspots that demonstrated the progression of a particular academic discipline. Figure 5 presented the top 50 references with the strongest citation bursts. It could be seen that the first reference to the literature began in 2009. The short duration of the citation literature suggested that this research field was poised for expansion and diversification in the coming years. Supplementary Table 3 showed the top 10 co-cited references. *Exosome-mediated transfer of mRNAs and microRNAs is a novel mechanism of genetic exchange between cells* are the most frequently cited articles (233 times).

## 4 Discussion

### 4.1 General information

Bibliometrics is fundamentally based on empirical statistical principles, including but not limited to Bradford's Law (17), Lotka's Law (18), Zipf's Law (19), and Price's Law (20). Bibliometric analysis software, which adheres to established scientific principles, offers an efficient and dependable method that provide a foundation for the analysis of this study. Our study strictly rigorously adhered to the principles of scientific statistics and provided new insights into the research of exosomes in aging, examining trends, emerging areas, research focal points, and collaborations among countries, institutions, and authors.

As shown in Figure 5 and Supplementary Table 3, during the period from 2007 to 2012, the role of exosomes as a medium for intercellular communication was established, which was distinguished from shedding microvesicles and apoptotic blebs (21). mRNA and miRNA contained in exosomes are increasingly recognized as potential biomarkers for diseases (22). Théry C indicated that the combination of exosomes from different sources with immunotherapies in clinical diseases is an important direction in the future research (23). Some studies had found the therapeutic effects of exosomes on neurological disorders (24, 25) and tumor (26). Meanwhile, the microvesicles had been shown to be distinct from exosomes, and the expression and role in immune regulation, inflammatory diseases, and cancer were increasingly being studied (27). From 2013 to 2017, one of the most significant achievements was the formulation of an experimental standard for extracellular vesicles by the International Society for Extracellular Vesicles (28). This development established a recognized standard for the research related to extracellular vesicles, enabling greater experiment standardization and ensuring research quality in the rapidly advancing field of extracellular vesicles. In the study of AD, various neurogenic plasma-derived exosomes could be assessed not only to predict progression of disease before the onset of AD (29), but also to potentially evaluate the effectiveness of clinical drugs (30). The focus on tumor cells was also a research hotspot. Masaki Takasugi found that exosomes released from senescent cells promote tumor proliferation through EphA2 (31). At the same time, an increasing number of studies had emphasized the importance of focusing on the microenvironment where the subject was situated and observing intercellular communication mediated by exosomes (32). From 2018 to 2023, a large number of reviews focused on the classification of exosomes and techniques of isolation (33, 34), including ultracentrifugation, immuno-affinity purification, microfluidics-based isolation techniques, size-based filtration, size-exclusion chromatography, and polymer precipitation, among others. Several studies had also indicated that exosomes from young individuals had the potential to delay aging. At the same time, the therapeutic role of MSC-derived exosomes in disease was emphasized. However, the application of MSC-derived exosomes encountered similar challenges related to isolation, quality control, and the reproducibility of efficacy that needed to be addressed (35).

In Figure 2A and Table 1, miRNAs were the most frequently appearing keywords besides exosomes and senescence. These results suggested that the study of exosomal miRNAs was very important in aging. In fact, exosomal miRNAs were involved in many processes leading to aging (Table 5). Autophagy played a crucial role in the degradation of abnormal proteins and was essential for the preservation of protein homeostasis. In the upstream, exosomal miRNAs were involved not only in autophagy but also in loss of proteostasis. Endothelial colony-forming cell-derived exosomal miR-21-5p regulated autophagic flux to promote vascular endothelial repair in atherosclerosis (36), but the effect of exosomes-derived miRNAs on autophagy was controversial. Another study showed that exosomal miR-4645-5p derived from BMSCs enhanced cellular autophagy, proliferation, and migration in diabetic murine models (37). It had been established that exosomal miRNAs played a significant role in the process of autophagy in aging. The influence of exosomal miRNAs on cellular aging was contingent upon the origin of the exosomes. Exosomal miR-3200-3p, originating from VEGFR suppressed tumor cells, had the capacity to target DDB1, thereby promoting T cell senescence (38). However, exosomal miR-214-3p derived from senescent osteoblasts promoted the acceleration of endothelial cell senescence to aggravate the osteoporosis (39). Nevertheless, the involvement of exosomal miRNAs in the aging process might be influenced by a range of factors, including the tissue source, cell type, physiological and pathological conditions. Consequently, additional research was required to elucidate these complexities.

**Table 5:** Exosomal miRNAs in hallmark of aging.

**Hallmark**	**miRNA**	**Species/Originate**	**Model**	**Target spot**	**References**
Disabled Macroautophagic and loss of proteostasis	miR-21-5p	Rats/Endothelial colony-forming cell	Atherosclerosis	SIPL1A2	(36)
	miR-83	Caenorhabditis elegans/Intestine and body wall muscle	Physiological aging	CUP-5/MCOLN	(40)
Genomic instability and cellular senescence	miR-767	Mice/Endothelial cells	Physiological skin aging	TAB1	(41)
	miR-146a-5p	Human/Hepatocellular Carcinoma Cells	Hepatocellular Carcinoma	IRF7	(42)
Epigenetic alterations	miR-21 and miR-217	Human/Umbilical vein endothelial cells and	Physiological aging	Unknown	(42–44)
	miR-195	Mice/Bone Marrow	Physiological aging	Tert	(45)
Mitochondrial dysfunction	miR-10a-5p	Rabbit/Plasma	Premature ovarian failure	BDNF	(46)
	miR-19b-3p	Human/Adipose	Abdominal aortic aneurysm	MST4/ERK/Drp1	(47)
Inflammation	miR-24-3p	Mice/umbilical cord MSCs	Physiological testicular aging	Unknown	(48)
	miR-212-3p	Mice/Osteoclast	Osteoarthritis	Smad2	(49)

### 4.2 Exosomes regulate the inflammatory response in aging and aging-related diseases

The phenomenon of elevated inflammatory markers in organisms and cells leads to the emergence of low levels of chronic pro-inflammatory states. Aging-related diseases are also exacerbated in inflammatory state, such as type 2 diabetes, cardiovascular diseases, and neurodegenerative diseases (50). Exosomes play a bi-directional role in regulating inflammation through intercellular communication, accelerating or delaying the process of aging and aging-associated diseases.

Exosomes regulate the initiation and progression of inflammation, particularly through exosomal miRNAs. For example, miR-19b, miR-21, miR-138, miR-146b, and miR-155 in endothelial cell-derived exosomes were closely related to vascular inflammation (51–54). It showed that IL-1β and TNF-α were increased in exosomes and contribute to the generation of inflammatory cascades (55). Based on these studies, it can be postulated that exosomes and exosomal miRNAs exacerbate cellular senescence and disease by influencing SASP-related inflammatory factors. In the same disease, exosomes from different sources exhibit varying effects on inflammation. Proinflammatory miRNAs of exosomes released by M1-type macrophages exacerbate myocardial injury in myocardial infarction mice (56). In contrast, exosomes from adipose-derived MSCs exhibit reduced levels of inflammatory markers as IL-1β, IL-6, TNF-α, and IFN-γ in patients with myocardial infarction (57). The beneficial anti-inflammatory effects in nervous system were obtained in exosomes and exosomal miRNAs, and these findings have significant implications for the management of neuroinflammatory diseases. Exosomes originating from human adipose-derived MSCs had been shown to suppress NF-κB and p38 mitogen-activated protein kinase, in addition to inhibiting the activation of microglia and macrophages (58). This mechanism is significant in mitigating neuroinflammation and facilitating functional recovery after brain injury. Moreover, exosomes derived from human adipose MSCs also alleviated microglia-mediated neuroinflammation through IRAK1/TRAF6 (59). Therefore, an effective way to delay aging and aging-related diseases is to fully utilize the anti-inflammatory effects of exosomes.

### 4.3 Exosomes have the potential to serve as biomarkers for cellular senescence

Cellular senescence is distinct from aging, characterized by the stable arrest of the cell cycle and concomitant alterations in morphology, structure, and function (60). The characteristics of cellular senescence can be observed at the early stage of embryonic development. Simultaneously, as the aging process progresses or diseases, senescent cells continue to accumulate within the body. Exosomes widely exist in almost all kinds of cells and affect the microenvironment through released miRNAs (61). It showed that exosomes and exosome-derived miRNAs exist a close relationship with the occurrence and progress of aging and aging-related diseases (62). It is important to mention that some of the exosome-derived miRNAs also have the potential to become the biomarkers of cellular senescence.

The study by Lehmann was significant because they found that aging of prostate cancer cells is closely linked to the role of P53 on exosomes in the extracellular environment (63). Numerous studies had shown that some exosomes and exosome-derived miRNAs accelerate cellular senescence. Exosomal miR-139-5p and senescent osteoblast-derived exosome-mediated miR-139-5p contributed to aging and cell apoptosis (64). In addition, inflammatory exosomes derived from senescent dendritic cells caused a large number of surrounding dendritic cells to aging through paracrine secretion (65). On the other hand, exosomes exhibit a delaying and inhibitory effects on cellular senescence. The mechanism of action was at least related to anti-inflammatory and anti-oxidative stress pathway. Dan Xu found that miR-22 was able to inhibit the development of breast cancer in mice, and this was attributed to miR-22 reactivating the cellular senescence program in cancer cells (66). MSCs-derived exosomes increased antioxidant capacity in senescent granulosa cells, thereby delaying cellular decline (67). Exosome-mediated adipose-derived MSCs enhanced the anti-inflammatory capacity of senescent cells in arthritis (68). Interestingly, exosomal miRNAs not only have the capacity to influence cellular senescence and aging-related diseases but also hold promises as biomarkers for SASP and aging-related diseases. For example, levels of exosomal miR-34a-5p and miR-183-5p increased with age (69, 70). Additionally, exosomal miR-24-3p in saliva from elderly patients showed the potential to serve as a biomarker of aging (7). Exosome-released miRNAs might serve as biomarkers to evaluate the quality of oocytes in the reproductive aging process (71).

### 4.4 Exosomes accelerate angiogenesis in aging and aging-related diseases

Regenerative medicine aims to find effective biological therapeutic methods to promote self-repair and regeneration of the organism. However, the efficiency of cell and tissue transplantation limits the development of regenerative medicine. The emergence of the paracrine hypothesis provides an opportunity to address this issue and attract much attention in regenerative medicine, especially regarding exosomes. According to the results of the bibliometric analysis, we focused on the therapeutic value and application of exosomes in angiogenesis.

Exosomes play a role in angiogenesis in different types of cancers. Mao revealed that exosomal miR-141 induces angiogenesis, playing a pro-cancer role in small cell lung cancer (72). Exosomal miR-210 not only exhibited elevated expression but also induced angiogenesis in lung cancer, breast cancer, and hepatocellular carcinoma (73–75). Several research advances had made on exosomes and exosomal miRNAs in the treatment of tumors. The direct transfection of exosomal miRNA-340 derived from young bone marrow stromal cells restored the anti-angiogenic effect of exosomes from old bone marrow stromal cells in bone tumors (76). Exosomal miR-944 derived from glioma stem cells delayed glioma progression by exerting angiogenesis inhibition (77). However, the utilization of exosomes is insufficient. The effective approach may involve regulating angiogenesis by targeting exosomes and exosomal miRNAs using drugs, biomaterials, and other methods.

### 4.5 Exosomes participate in the regulation of insulin resistance

IR is a decrease in the body's efficiency in promoting glucose uptake and utilization by insulin, which is an important feature of diabetes mellitus. Immune aging is a manifestation of aging, and the process of immune aging affecting insulin metabolism is highly correlated with various SASP inflammatory factors (78).

Mitochondrial dysfunction represents a significant factor in the aging process; however, exosomes derived from macrophages had been shown to adversely affect mitochondrial function in murine models of type 2 diabetes (79). Li showed that miR-27-3p from M2-derived exosomes exacerbated the development of IR and diabetes through mitophagy (80). The different effects on IR may be related to the source of exosomes and the function of different macrophages. The exosomes from Natural killer cells improved IR, and the exosomal miR-1249-3p improved cellular insulin sensitivity (81). Su showed that the level of exosomal miR-29b-3p derived from bone marrow MSCs of aging mice was closely related to Sirt1, and inhibition the level of exosomal miR-29b-3p improved the IR associated with aging (4). Jalabert found that the changes in exosomal levels in mice might be partially closely related to PPARγ, resulting in modifications to IR (82). The AMP-activated protein kinase (AMPK) pathway enhanced insulin sensitivity by inhibiting antagonistic insulin signaling (83), and mammalian target of rapamycin (mTOR) was involved in glucose metabolism and angiogenesis (84). Therefore, exosomes derived from different cells play a crucial role in regulating IR by facilitating the transfer of nuclear materials or transmitting intercellular signals. This process ultimately alleviates IR-induced aging and aging-related diseases.

### 4.6 Exosomes prove beneficial for the diagnosis and treatment of Alzheimer's disease

AD is one of the most common neurodegenerative diseases, and its incidence is increasing every year as the world's population ages. The nervous system is capable of producing and releasing exosomes, which contributes to synaptic plasticity, myelination, neurogenesis, and the regulation of neuroinflammation (85). Exosomes transmit information between neurons and neuronal-glial cells and play a role in the development of AD, leading to the accumulation of proteins such as amyloid beta (Aβ) and tau.

Extensive studies have been conducted on the treatment of AD with favorable results, such as stem cell-derived exosomes and exosomal miRNAs. MSC-secreted exosomes effectively reduced the expression of the inflammatory mediators TNF-α, IL-1β, and IL-6 in AD mice (86). Another study showed that MSCs-derived exsomes enhanced resistance to soluble oligomers of the Aβ-induced oxidative stress and synaptic damage in AD rats (87). The ability of MSCs to internalize soluble Aβ oligomers provides significant research directions and value to MSC-derived exosomes for the treatment of AD. Besides MSCs-derived exosomes, exosomes from other types of stem cells exhibit similar capabilities. Exosomal miRNAs derived from neuronal stem cells have been shown to enhance synaptic resistance to amyloid oligomers (88). The research on using exosomes as carriers for drug delivery is also increasing. For example, Ashok Iyaswamy ameliorated cognitive decline in AD mice using exosomes derived from hippocampal neuronal cells overexpressing Fe65 as a carrier for the targeted delivery of Corynoxine-B (89). Saliva-derived exosomes from AD patients had the potential to serve as new biomarkers for AD. The expression of neurogenic exosomes was elevated in the saliva of AD patients (90). Several clinical research has demonstrated changes in the expression of miRNAs in salivary-derived exosomes in AD patients, such as exosomal miR-342-3p and miRNA-485-3p (91, 92). Although exosomes in the cerebrospinal fluid of AD patients appear to be more sensitive, exosomes and exosomal miRNAs derived from saliva are more easily accessible. It is important to emphasize the role of exosomes from different sources for AD treatment, particularly from stem cells.

### 4.7 The hotspots and promises of exosomes for aging and aging-related diseases

1. Currently, the most important research direction in exosomes is to find easier and more effective methods for exosome isolation and purification.

2. Exosomes have emerged as potential biomarkers for detecting and diagnosing certain aging-related diseases. Nonetheless, current research lacks systematicity and breakthrough findings. Future research should focus on enhancing the exploration of exosomes as biomarkers of aging, which can hold promise for effectively evaluating the aging process and the risk of aging-related diseases.

3. The current hot direction is the role of exosomes in the pathogenesis of aging-related diseases, such as neurodegenerative diseases, cardiovascular diseases, and tumors. It is indispensable to study the molecular mechanisms of exosomes production and release in aging and aging-related diseases. At the same time, researchers should also focus on the specific mechanisms of exosomes in intercellular communication and tissue microenvironment.

4. The utilization of exosomes as therapeutic drug delivery vehicles for the treatment of aging-related diseases represents a promising avenue for future research. However, the drug delivery efficiency of exosomes is poor at present. The combination of biomaterials and exosomes is one of the viable options to enhance the efficiency of exosomes therapy. In future research, we should pay more attention to the development and application of biomaterials combined with exosomes.

5. SASP plays a significant role in aging and aging-related diseases. However, it is uncertain whether exosomes play a therapeutic role in aging and aging-related diseases by intervening SASP. Therefore, it is necessary to strengthen the research on the synergistic effect of human exosomes and SASP to change the cellular microenvironment and affect the proliferation and differentiation of adjacent cells.

6. There is no definitive conclusion about the effects of exosomes on cellular senescence programs. In the future, the relationship between exosomes and aging processes such as telomere shortening, DNA damage, decreased mitochondrial function, and lipid metabolism should be further investigated.

7. Stem cell research is a significant focus in the field of regenerative medicine, and extracellular vesicles also possess regenerative capabilities similar to stem cells. To delay aging and treat aging-related diseases, it is necessary to optimize the regenerative capacity of extracellular vesicles from different sources, and to apply extracellular vesicle therapy in clinical settings.

## 5 Strengths and limitations

This is the first study to perform a visual analysis of exosomes in aging. CiteSpace, VOSviewer and Bibliometrix were used for clustering in order to make the derived analysis data more intuitive. Through the bibliometric retrieval and data analysis of this study, researchers can efficiently comprehend the fundamental concepts, research basis, processes, hotspots, and trends on “exosomes in aging.” In addition, it can also provide a reference for scholars to search for literature, research topics, and journal submissions.

However, there are also limitations to our investigation. CiteSpace, VOSviewer, and Bibliometrix are only bibliometric analysis software and cannot completely replace systematic retrieval systems. Documents were retrieved from January 1, 2007, to December 18, 2023, and the article was written in English. Articles published in this area before 2007 and after 2024 were excluded from our analysis. Despite these limitations, we don't believe that they significantly impact the overall context and trend of “exosomes in aging.” Therefore, this study still holds reference value for subsequent research.

## 6 Conclusion

The research on exosomes in aging demonstrates a steadily rising trend, with increasing number of scholars, institutions, and countries joining the effort. They have consistently published high-quality research results in internationally influential journals and will continue to advance the development of this field. However, the research among different countries, institutions, and scholars is relatively isolated and lacks stable exchange and collaboration. The exchange and cooperation among countries, institutions, and scholars should be continuously strengthened. The exploration of this area should focus more on the fundamental and clinical transformations of the research. The current and future research is centered on investigating the role of exosomes in the process of aging and aging-related diseases, as well as exploring how interventions involving exosomes can potentially mitigate the progression of aging and associated conditions.
