# Supplementary material for: A bibliometric analysis of exosomes in aging from 2007 to 2023
Source: Front Med (Lausanne). 2025 Jan 22;11:1488536. doi: 10.3389/fmed.2024.1488536 (PMC11794001; doi:10.3389/fmed.2024.1488536)
Supplement: Supplementary file 1 [file Data_Sheet_1.docx]

Supplementary

| **Cluster** | **Keywords** |
| --- | --- |
| Red | activation, angiogenesis, apoptosis, bone-marrow, cellular senescence, damage, delivery, differentiation, endothelial cells, exosomes, in-vitro, injury, macrophages, mesenchymal stromal cells, mesenchymal stem cells, migration, osteogenic differentiation, osteoarthritis, oxidative stress, pathway, progenitor cells, proliferation, regeneration, regenerative medicine, repair, secretome, senescence, skin, stem cells, stromal cells, therapy, tissue, transplantation |
| Green | alpha-synuclein, Alzheimer's disease, amyloid-beta, association, blood exosomes, brain, cerebrospinal-fluid, cognitive impairment, dementia, dysfunction, inhibition, mice, microglia, model, mouse model, neurodegeneration, Parkinson’s disease, protein, receptor, survival |
| Bule | Biomarkers, blood, cancer, cell, circulating miRNAs, diagnosis, expression, gene-expression, genes, growth, identification, mRNA, metastasis, plasma, progression, proteomics, RNA, serum |
| Yellow | adipose-tissue, aging, communication, disease, inflammation, insulin-resistance, metabolism, microparticles, obesity, pregnancy, release, risk, roles |
| Violet | Autophagy, biogenesis, cell-derived, dendritic cells, extracellular vesicles, in-vivo, mechanism, membrane-vesicles, microvesicles, pathogenesis, secretion, transferrin receptor, vesicles |

**Supplementary Table 1.** Co-occurrence analysis of keywords in the 3 clusters.

| **NO** | **Co-cited authors** | **Count (%)** | **Centrality** | **Representative articles** |
| --- | --- | --- | --- | --- |
| 1 | Thery C | 466 | 0.21 | Minimal information for studies of extracellular vesicles 2018 (MISEV2018): a position statement of the International Society for Extracellular Vesicles and update of the MISEV2014 guidelines |
| 2 | Valadi H | 230 | 0.06 | Exosome-mediated transfer of mRNAs and microRNAs is a novel mechanism of genetic exchange between cells |
| 3 | Raposo G | 200 | 0.05 | A brief history of nearly EV-erything - The rise and rise of extracellular vesicles |
| 4 | Zhang Y | 179 | 0 | Hypothalamic stem cells control ageing speed partly through exosomal miRNAs. |
| 5 | Colombo M | 172 | 0.01 | Biogenesis, secretion, and intercellular interactions of exosomes and other extracellular vesicles |
| 6 | Kalluri R | 170 | 0.02 | Summary of the ISEV workshop on extracellular vesicles as disease biomarkers, held in Birmingham, UK, during December 2017 |
| 7 | Van Niel G | 159 | 0.04 | Shedding light on the cell biology of extracellular vesicles |
| 8 | YÁÑEZ-MÓ M | 127 | 0.01 | A simple immunoassay for extracellular vesicle liquid biopsy in microliters of non-processed plasma |
| 9 | Alvarez-Erviti L | 104 | 0.05 | Exosomes and autophagy: coordinated mechanisms for the maintenance of cellular fitness |
| 10 | Kowal J | 103 | 0.22 | Proteomic comparison defines novel markers to characterize heterogeneous populations of extracellular vesicle subtypes. |

**Supplementary Table 2** TOP 10 Co-cited-authors and their published articles.

| **NO** | **Title** | **First author** | **Source** | **Strength** | **Year** |
| --- | --- | --- | --- | --- | --- |
| 1 | The biology, function, and biomedical applications of exosomes | Kalluri R | *Science* | 29.95 | 2020 |
| 2 | Extracellular vesicles: Exosomes, microvesicles, and friends | Raposo G | *Journal of Cell Biology* | 21.98 | 2013 |
| 3 | Biogenesis, Secretion, and Intercellular Interactions of Exosomes and Other Extracellular Vesicles | Colombo M, | *Annual Review of Cell and Developmental Biology* | 21.65 | 2014 |
| 4 | Biological properties of extracellular vesicles and their physiological functions | Yáñez-Mó M | *Journal of Extracellular Vesicles* | 19.91 | 2015 |
| 5 | Identification of preclinical Alzheimer's disease by a profile of pathogenic proteins in neurally derived blood exosomes: A case-control study | Fiandaca MS | *Alzheimer's & Dementia* | 13.08 | 2015 |
| 6 | Communication by Extracellular Vesicles: Where We Are and Where We Need to Go | Tkach M | *Cell* | 10.37 | 2016 |
| 7 | Membrane vesicles as conveyors of immune responses | Théry C | *Journal of Extracellular Vesicles* | 10.18 | 2009 |
| 8 | Overview of Extracellular Vesicles, Their Origin, Composition, Purpose, and Methods for Exosome Isolation and Analysis | Doyle LM | *Cells* | 9.69 | 2019 |
| 9 | Tumour exosome integrins determine organotropic metastasis | Hoshino A | *Nature* | 9.18 | 2015 |
| 10 | Shedding light on the cell biology of extracellular vesicles | van Niel G | *Nature reviews molecular cell biology* | 8.95 | 2018 |

**Supplementary Table 3** Top 10 co-citation of cited references on “exosomes in aging”


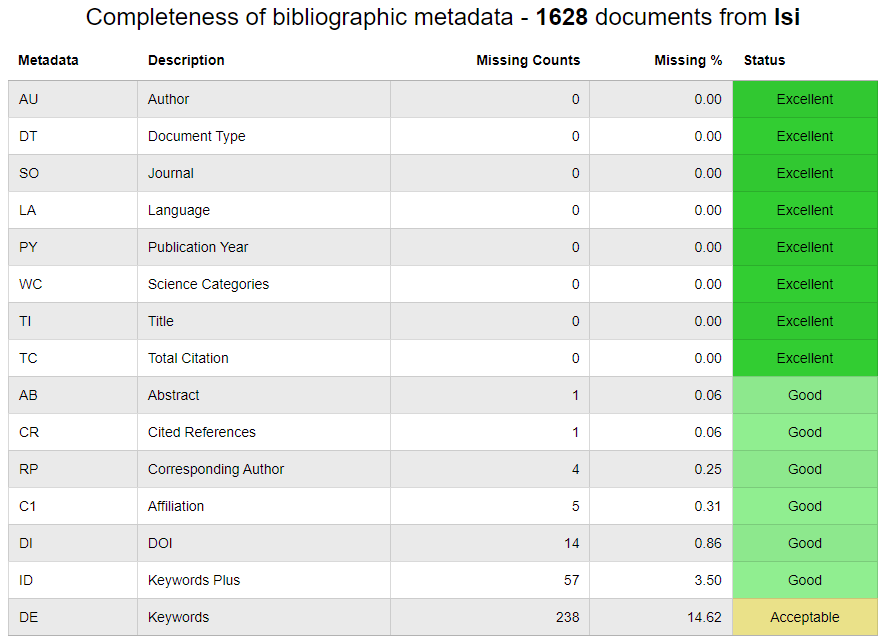


**Supplementary** **Figure 1** The evaluation results for metadate in Bibliometrix.


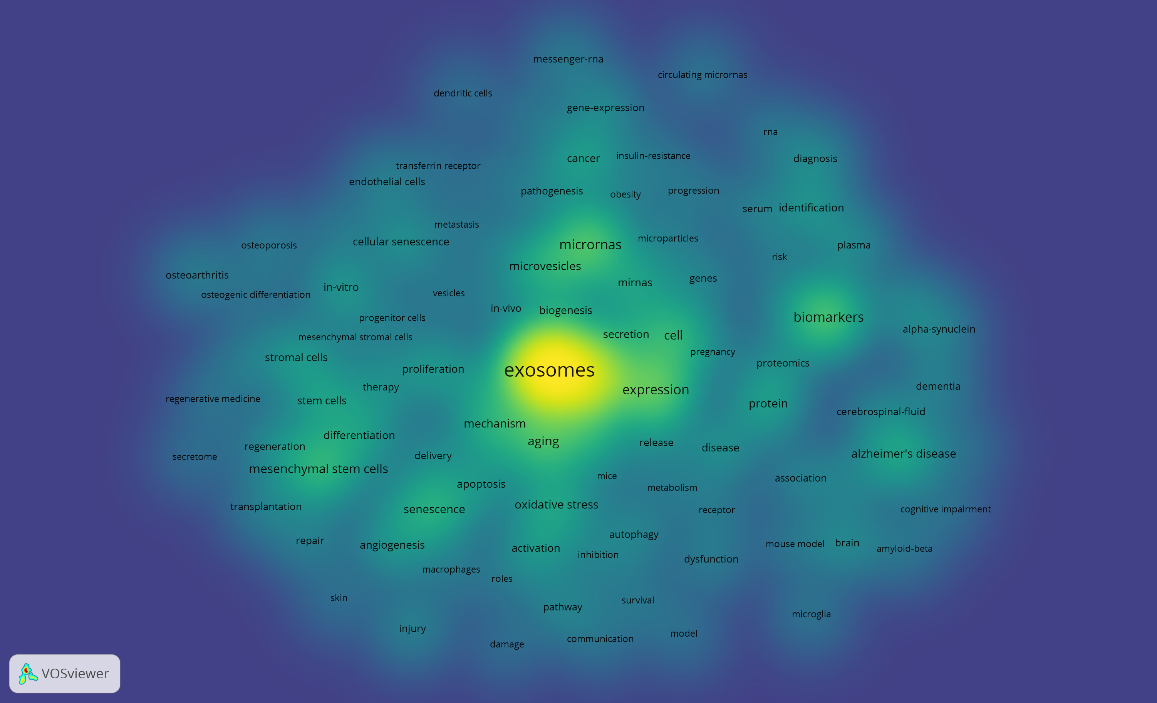


**Supplementary Figure 2** The density map of keyword co-occurrence related to exosomes in aging.


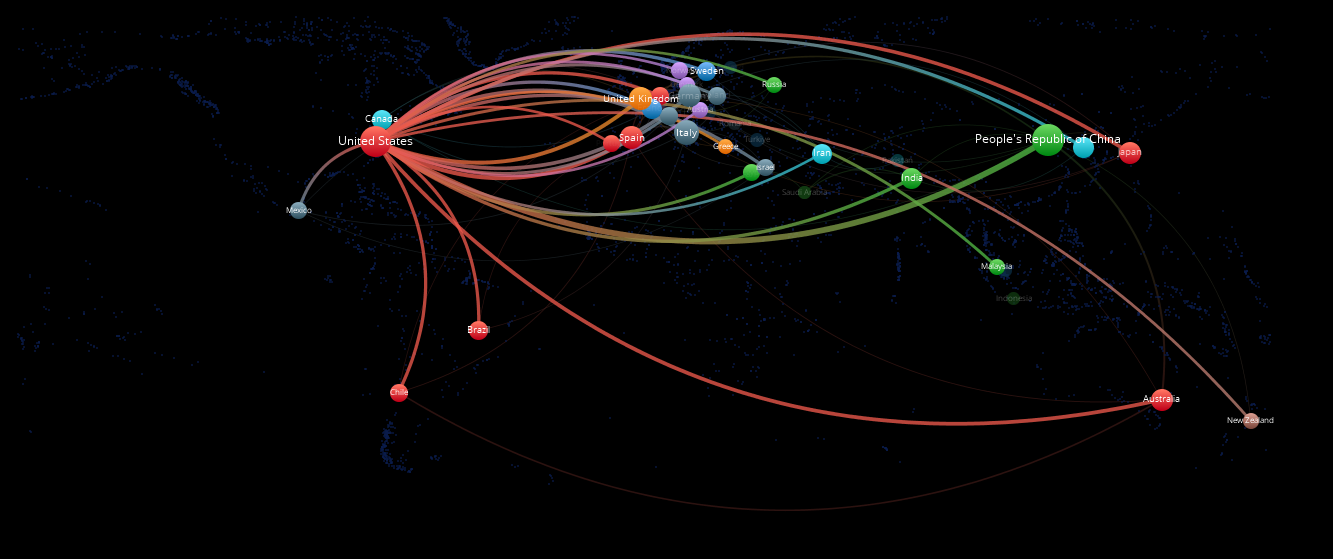


**Supplementary Figure 2** the United States cooperative network in exosomes in aging.


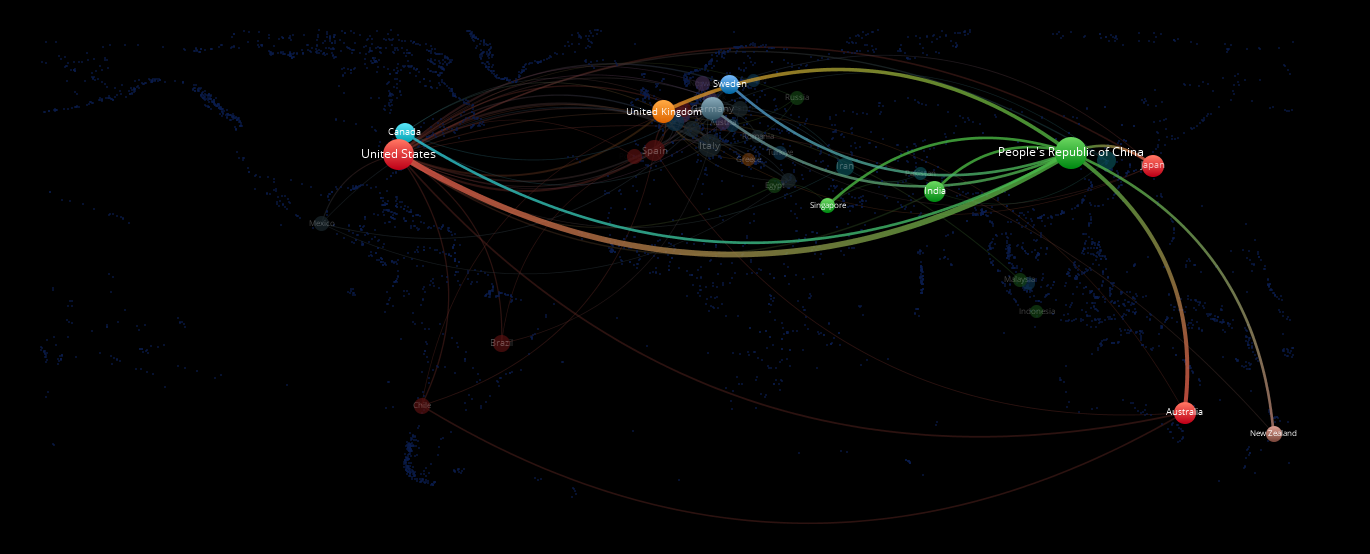


**Supplementary Figure 3** China cooperative network in exosomes in aging.
